# ASO Author Reflections: What is the Oncologically Acceptable Surgical Waiting Time in Patients with cT1bN0M0 Squamous Cell Carcinoma of the Thoracic Esophagus?

**DOI:** 10.1245/s10434-026-20008-5

**Published:** 2026-06-22

**Authors:** Yutaka Miyawaki, Hisashi Fujiwara, Shigeo Haruki, Hiroshi Sato

**Affiliations:** 1 Department of Gastroenterological Surgery, Saitama Medical University International Medical Center, Saitama, Japan; 2 Department of Gastroenterological Surgery, Institute of Science Tokyo, Tokyo, Japan; 3 Department of Biostatistics, Graduate School of Medicine, Saitama Medical University, Saitama, Japan

## Past

It is clear that malignant tumors progress over time. Therefore, even in cases of thoracic esophageal squamous cell carcinoma (ESCC) classified as cT1bN0M0—which is considered to be a relatively early stage—prompt initiation of treatment is recommended. Although some studies suggest that prolonged surgical waiting time is associated with poorer survival outcomes,^1^ others report no significant effect of diagnostic-to-surgical delay on long-term survival,^2^ leaving the impact unclear.

However, most reports have focused on cases of advanced-stage disease that underwent preoperative therapy followed by surgery. Reports on cT1bN0M0 esophageal cancer—which is an indication for upfront surgery—have been limited to adenocarcinoma, the major subtype of esophageal cancer in Western countries. Consequently, the association between surgical waiting time and postoperative prognosis in ESCC remains unclear.

## Present

Our multicenter retrospective study included 160 patients with cT1bN0M0 ESCC undergoing subtotal esophagectomy with lymphadenectomy at seven Japanese institutions between 2008 and 2021.^3^ Receiver operating characteristic analysis identified the optimal waiting time cutoff predicting recurrence.

Receiver operating characteristic analysis identified 66.5 days as the optimal cutoff value. The ‘long waiting’ group (≥67 days) had significantly worse 3-year recurrence-free survival (87.9% vs. 73.5%, log-rank* P*<0.01; hazard ratio 2.71; 95% confidence interval 1.29–5.70, *P*<0.01), whereas cancer-specific survival showed no significant difference. Multivariate analysis identified prolonged waiting time, pathological T2 or deeper invasion, and lymph node metastasis as independent predictors of recurrence, with longer delays linked to increased systemic recurrence beyond 2 years postoperatively.

## Future

The cutoff value of 66.5 days established in our study should be regarded as an exploratory result rather than definitive because of the limitations of statistical power resulting from the single-center retrospective observational nature of the study and the small number of outcome events, . However, using optimized preoperative waiting times as a guideline for determining the timing of surgery may lead to a reduction in the risk of recurrence. Ultimately, validation in a larger cohort is necessary to define an acceptable preoperative waiting period.
